# Repercussions of Caregiving on Caregivers of Stroke Survivors: A Cross-Sectional Study

**DOI:** 10.7759/cureus.51250

**Published:** 2023-12-28

**Authors:** Ravi Gaur, Satyasheel S Asthana, Nitesh M Gonnade, Amit Ranjan, Dhaval Morvadiya

**Affiliations:** 1 Physical Medicine and Rehabilitation, All India Institute of Medical Sciences, Jodhpur, Jodhpur, IND; 2 Physical Medicine and Rehabilitation, All India Institute of Medical Sciences, Raebareli, Raebareli, IND; 3 Physical Medicine and Rehabilitation, All India Institute of Medical Sciences, Gorakhpur, Gorakhpur, IND

**Keywords:** caregiver strain, rehabilitation, modified caregiver strain index, stroke survivor, stroke caregiver

## Abstract

Background: Stroke is one of the most common causes of disability. Stroke survivors may have a wide variety of sensorimotor, cognitive, perceptual, and behavioral dysfunctions. The majority of long-term care for stroke survivors in residential settings is provided by informal caregivers, such as family members. This study was conducted to assess the burden of caregiving on caregivers of stroke survivors.

Materials and methods: This cross-sectional study was conducted by the Department of Physical Medicine and Rehabilitation in a tertiary care institute in Western India. Patients were evaluated for inclusion and exclusion criteria. Caregiver strain among caregivers was assessed using the Modified Caregiver Strain Index Questionnaire (MCSI). The Katz index was used to assess activities of daily living.

Results: The inclusion and exclusion criteria were fulfilled by 125 primary caregivers of stroke patients. Among stroke survivors, the majority were male (57.6%), and caregivers were wives of stroke survivors (28.8%). There was a significant statistical difference in the median of the modified caregiver strain index when the stroke survivor was male (p=0.034), fully dependent (p<0.001), and had a hemorrhagic stroke (p<0.001). There was no significant statistical difference in the median of the MCSI based on the sex of caregivers (p=0.928). There was a positive correlation between the age of the patient and MCSI (r=0.373, p<0.001). No correlation was found between the MCSI and age of caregivers (r=-0.108, p=0.230) and duration of stroke (r=-0.089, p=0.321).

Conclusion: The findings in our study provide evidence that caregivers of stroke survivors experience significant levels of strain. It is desirable to recognize caregiver strain during the rehabilitation of stroke survivors and manage it appropriately.

## Introduction

Stroke is one of the most common causes of mortality worldwide [1]. It is also one of the most common causes of disability in adults [2]. It is associated with a wide variety of sensorimotor, cognitive, perceptual, and behavioral dysfunctions [3]. These impairments may limit the ability of stroke survivors to independently perform their activities of daily living. This disability also restricts effective participation in family and social roles.

The majority of long-term care for stroke survivors in residential settings is provided by informal caregivers, such as family members. The psychological and physical health of caregivers, who offer both physical and emotional support to stroke survivors on a daily basis, is adversely affected. Caregiver burden is a term used to describe the weight or load carried by caregivers as a result of adopting the caregiving role [4].

Stroke onset is sudden, and there is a change in the role of family members towards stroke survivors. Caregivers are often ill-prepared for these sudden life changes [5,6]. These caregivers play an important role in post-stroke rehabilitation of stroke survivors, which is often a neglected domain. Stroke survivor’s caregiver experience lots of strain. This strain can be psychological, physical, financial, and social [7,8]. Therefore, this study was conducted to assess the burden of caregiving on caregivers of stroke survivors.

## Materials and methods

Setting: Out-patient Department of Physical Medicine and Rehabilitation in All India Institute of Medical Sciences, Jodhpur, Rajasthan, India.

Type of study: A cross-sectional study

Ethical clearance: All procedures were carried out in accordance with the Helsinki Declaration of 1975, as amended in 2000, and the ethical guidelines established by the relevant committee on human experimentation (regional or institutional) [9]. Institutional Ethical Clearance (AIIMS/IEC/2020/3207) was taken prior to the enrollment of patients in the study. Patients were evaluated for inclusion and exclusion criteria. Informed consent was also taken.

Subjects: Caregivers of stroke survivors and stroke survivors

Inclusion criteria: Caregivers of stroke survivors of more than one-month duration and caregiver age of >18 years.

Exclusion criteria: Private caregivers; caregivers not willing to take part in the study; caregivers of stroke survivors with amputation of any limb, spinal cord injury, and cancer/metastasis; caregivers of stroke survivors whose age is more than 70 years; and caregivers taking any mental healthcare.

Sampling and study duration: Consecutive sampling was done. Patients were enrolled in the study from November 2020 to May 2021.

Interventions: Questionnaire-based. The Modified Caregiver Strain Index (MCSI) was used to evaluate the strain on caregivers of stroke survivors. It is an easy-to-use self-administered questionnaire. This tool is used to assess the strain of caregiving on caregivers with chronic medical conditions (stroke, dementia, and cancer) [10]. It is a 13-item questionnaire with a minimum score of 0 and a maximum score of 26. The each of 13 items is divided into three responses: 0 means no problem, 1 means a problem occurred sometimes, and 2 means a problem is present at all times. High scores suggest a high degree of caregiver strain [11,12]. The tool is valid and has internal consistency, which is reported to be 0.90 [11].

The Katz index was used to group patients as fully dependent, partially dependent, and independent in activities of daily living (ADL). In the Katz index, the patient’s performance is assessed in various ADLs that include activities such as eating, incontinence, bathing, dressing, toileting, and transferring activities. The Katz index has a minimum score of 0 (completely dependent) and a maximum score of six (completely independent). The Katz index is a valid tool, with an internal consistency of 0.94 in assessing ADL [13-15].

Statistical analysis: The continuous variables are presented as mean with standard deviation. The non-continuous variable is presented as frequency tables (in number and percentage). The Shapiro-Walik test was applied to evaluate the distribution of continuous variables. The data were not normally distributed. P-values for the age of patients (in years), duration of stroke (in months), age of caregiver (in years), and MCSI were found to be less than 0.001. Therefore, a non-parametric test was used. The comparison of the MCSI between variables was done using the Mann-Whitney test. The Spearman correlation test was used to assess the correlation. Data were analyzed using Statistical Product and Service Solutions (SPSS, v23) (IBM SPSS Statistics for Windows, Armonk, NY). For a confidence interval of 95%, a p-value of less than 0.05 was considered statistically significant.

## Results

During the duration of the study, a total of 180 stroke survivors visited the outpatient department of Physical Medicine and Rehabilitation for Neuro-rehabilitation with their caregivers. Fifty-five caregivers were not included in the study. Among the exclusions, six caregivers refused to take part in the study, four were less than 18 years of age, 13 caregivers were private caregivers (paid), and five caregivers were taking treatment from a psychologist/psychiatrist (before the start of caregiver responsibility; no caregiver started any such treatment after the start of caregiving to stroke survivors). Six stroke survivors were more than 70 years old, and the stroke duration of 21 stroke survivors was less than one month. They were also excluded. A total of 125 caregivers fulfilled the inclusion and exclusion criteria of the study and were included in the data analysis.

Characteristics of stroke survivors

The mean age of stroke survivors was 57.07±10.12 years (range: 23-70 years) with a mean duration of stroke of 14.96±10.45 months (range: five months to 68 months). Among the stroke survivors, the majority were male (57.6%) and married (88.8%). Ischemic stroke (57.6%) was more common in stroke survivors. Moreover, 75.2% were right-hand dominant. The most common presentation was with right hemiplegia (48.8%), followed by left hemiplegia (40.8%) (Table 1). According to the score of the Katz index, 54.4% of stroke survivors scored zero (fully dependent), while 45.6% scored between 1 and 5 (partially dependent). None of the patients scored 6 (fully independent).

**Table 1:** Characteristics of stroke survivors and caregivers

Characteristic	Sub-group	Frequency	Percent
Gender of Patient	Male	72	57.6
	Female	53	42.4
Gender of Caregiver	Male	52	41.6
	Female	73	58.4
Marital Status of Patient	Married	111	88.8
	Not Married	9	7.2
	Widowed	3	2.4
	Divorced	2	1.6
Marital Status of Caregiver	Married	95	76.0
	Not Married	27	21.6
	Widowed	2	1.6
	Divorced	1	0.8
Presentation of Stroke	Right Hemiplegia	61	48.8
	Left Hemiplegia	51	40.8
	Monoplegia	7	5.6
	Quadriplegia	6	4.8
Dependency	Fully Dependent	68	54.4
	Partially Dependent	57	45.6
Dominant Hand	Right	94	75.2
	Left	31	24.8
Type of Stroke	Ischemic	72	57.6
	Hemorrhagic	53	42.4

Characteristics of caregivers

**Figure 1:**
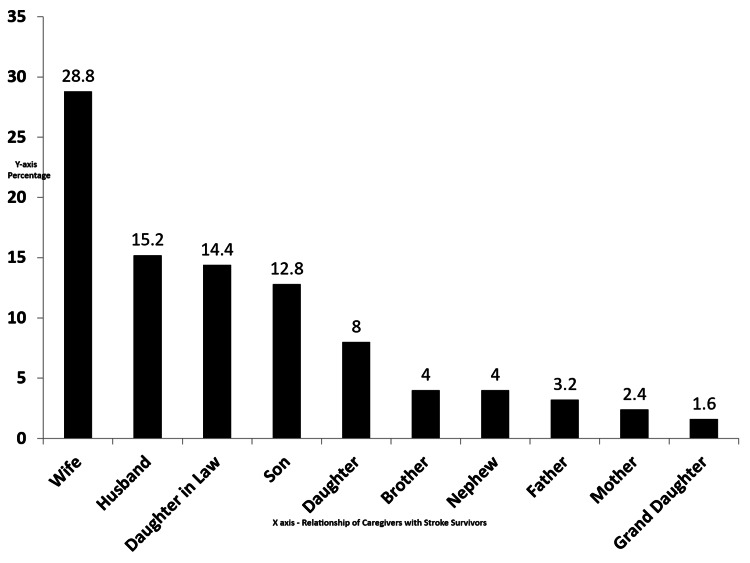
Distribution of caregivers in percentages according to their relationship with stroke survivors

The mean age of caregivers was 44.04±14 years (range: 19-69 years). Females (58.4%) were most commonly involved in the caregiving of stroke survivors, and 76% of caregivers were married. Among the caregivers, the wife of a stroke survivor (28.8%) was most common followed by the husband (15.2%), daughter-in-law (14.4%), and son of a stroke survivor (12.8%) (Figure 1).

Specifically, 61.6% of caregivers were employed. Self-employed caregivers were doing various businesses (grocery stores, beauty parlors, part-time tuitions, renting property, advisory, etc.) for a steady source of income, and 34.4% (43) of caregivers were not employed. Among the unemployed caregivers, 29.9% (12) were students, 18.6% (eight) were looking for jobs, and 53.48% (23) of them were not looking for jobs due to the financial stability of the family (supported by other family members or pension of stroke survivors as the source of income). Additionally, 4.8% of caregivers lost their jobs, while 8% of caregivers changed their jobs to ease into caregiving for their loved ones.

MCSI

The mean MCSI was 12.09±4.57, with a range of 2-23. None of the caregivers reported zero strain. Severe burden of caregiving was reported in 16.8% of caregivers, while moderate and mild strain were reported in 53.6% and 29.6% of caregivers, respectively.

MCSI questionnaire responses are tabulated in Table 2. The following were the most commonly reported by caregivers on a daily basis: feeling completely overwhelmed (48%), caregiving is inconvenient (40%), and sleep is disturbed (37.6%). Caregivers had to, sometimes, do work adjustments (56%) or make emotional adjustments (48.8%). Moreover, 43.2% of caregivers reported that sometimes they feel caregiving is a physical strain, while 32% felt it on a regular basis.

**Table 2:** Caregiver responses to the Modified Caregiver Strain Index n is the total number of responses by caregivers within each item of the Modified Caregiver Strain (total number (percentage, %)).

Items in Modified Caregiver Strain Index	No (n)	Yes, sometimes (n)	Yes, on a regular basis (n)
My sleep is disturbed	24 (19.2 %)	54 (43.2 %)	47 (37.6 %)
Caregiving is inconvenient	40 (32 %)	35 (28 %)	50 (40 %)
Caregiving is a physical strain	31 (24.8 % )	54 (43.2 %)	40 (32 %)
Caregiving is confining	42 (33.6 %)	50 (40 %)	33 (26.4 %)
There have been family adjustments	49 (39.2 %)	45 (36 %)	31 (24.8 %)
There have been changes in personal plans	50 (40 %)	52 (41.6 %)	23 (18.4 %)
There have been other demands on my time	52 (41.6 %)	42 (33.6 %)	31 (24.8 %)
There have been emotional adjustments	27 (21.6 %)	61 (48.8 %)	37 (29.6 %)
Some behavior is upsetting	59 (47.2 %)	44 (35.2 %)	22 (17.6 %)
It is upsetting to find the person I care for has changed so much from his/her former self	72 (57.6 %)	30 (24 %)	23 (18.4 %)
There have been work adjustments	38 (30.4 %)	70 (56 %)	17 (13.6 %)
Caregiving is a financial strain	46 (36.8 %)	52 (41.6 %)	27 (21.6 %)
I feel completely overwhelmed	24 (19.2 %)	41 (32.8 %)	60 (48 %)

Comparisons of the MCSI

Intra-categorical comparisons of the MCSI are tabulated in Table 3. There was a significant statistical difference in the median of the MCSI when stroke survivors were male (p=0.034). Caregiving of stroke survivors who were fully dependent on caregivers also showed a significant statistical difference in median (p<0.001). A significant difference in the median MCSI was also seen in hemorrhagic stroke (p<0.001). There was no significant statistical difference in the median MCSI based on the gender of caregivers (p=0.928). Caregiving of male, fully dependent stroke survivors and stroke survivors with hemorrhagic stroke was found to be more cumbersome. Caregivers, whether male or female, felt similar levels of strain.

**Table 3:** Intra-categorical comparisons of the Modified Caregiver Strain Index SD = Standard deviation; n = Number of caregivers in category; * Mann-Whitney test

Variable	Category	n	Mean±SD	Median	Mean Rank	P value^*^
Sex of Caregiver	Male	52	12.11±4.80	11	63.35	0.928
	Female	73	12.08±4.43	11	62.75	
Sex of Patient	Male	72	12.90±4.58	11.5	68.87	0.034
	Female	53	11.00±4.35	10	55.03	
Dependency	Complete	68	13.31±4.52	12	73.60	<0.001
	Partial	57	10.64±4.22	10	50.35	
Type of Stroke	Ischemic	72	10.37±3.94	10	48.81	<0.001
	Hemorrhagic	53	14.43±4.33	13	82.28	

Correlation of the MCSI

There was a positive correlation between the age of the patient and MCSI (r=0.373, p<0.001). This suggests that the burden of caregiving increases with the age of the patient. No correlation was found between the MCSI and age of caregivers (r=-0.108, p=0.230) and the duration of stroke (r=-0.089, p=0.321) (Table 4).

**Table 4:** Correlations of the Modified Caregiver Strain Index † Spearman correlation test

Variable	No. of patients	Correlation coefficient	P-value†
Age of Caregiver	125	-0.108	0.230
Age of Patient	125	0.373	<0.001
Duration of Stroke	125	0.089	0.321

## Discussion

Stroke is a cerebrovascular disease that is devastating for both patient and family. Stroke can lead to a wide variety of outcomes, which can range from complete recovery to severe disability or death. Morbidity due to stroke can drastically impact the activity of daily living of stroke survivors. Family members play an important role in the rehabilitation of stroke survivors. Caregivers of stroke survivors experience a lot of strain, which can be physical, mental, or financial. Caregivers are at increased risk of depression, fatigue, changes in social dynamics, and physical health-related issues. High stress in caregivers can drastically impact the rehabilitation of stroke survivors [16]. This stress significantly affects the lives of caregivers [17].

Our study has female predominance in caregiving. Wives of stroke survivors were the most common caregivers, followed by husbands, daughters-in-law, and sons of stroke survivors. Female predominance in caregiving is supported by many studies [2,18-21]. This may be due to cultural practices in India [18,22]. However, some studies also reported male predominance in caregiving in stroke survivors [10,17,23].

Broadly, stroke can be hemorrhagic or ischemic. Hemorrhagic stroke is associated with high mortality and morbidity [24]. In the long term, the functional and clinical outcome of hemorrhagic stroke is not as good as ischemic stroke [25]. In our study, strain in caregivers of stroke survivors with hemorrhagic stroke was significantly higher. Caregivers of fully dependent stroke survivors also experienced a significantly higher burden of caregiving. Blake et al. reported a significant relationship between caregiver strain and the level of disability in ADL [26]. The burden of caregiving is higher in caregivers of fully dependent stroke survivors [27]. Strain related to caregiving may depend on the level of patient dependency on caregivers, duration of illness, cognition of patient, and financial status [17].

A cross-sectional study conducted by Kaur et al. in India concluded that caregiver strain significantly affects the life of caregivers. The MCSI showed that the most common persistent problems were disturbed sleep (92.5%), inconvenience of caregiving (88.7%), and feeling overwhelmed (77.4%) [17]. In our study, the most common persistent problems were feeling overwhelmed (48%), inconvenience of caregiving (40%), and disturbed sleep (37.6%).

Ain et al. in 2014 did not find any significant difference in the MCSI between genders of caregivers [10]. Blake et al. also did not find a significant difference in caregiver strain based on the gender of caregivers [26]. Similar findings were also reported by Sardar et al. in 2022 [27]. These findings are similar to our study, which did not find any statistical difference in the MCSI between male and female caregivers. Caregiver strain felt by caregivers of stroke survivors is not dependent on the gender of caregivers.

Fuh et al. gave two hypotheses to explain the burden of caregiving. The “Wear and tear hypothesis” states that, as the duration of the disease increases, the burden of caregivers also increases. The second hypothesis, the “adaptation hypothesis,” states that, as the duration of the disease increases, the burden on caregivers decreases as caregivers learn to tolerate the burden of caregiving [28]. Blake et al. reported that the caregiver burden may increase with the duration of stroke, supporting the wear and tear hypothesis [26]. In our study, we did not find any correlation between the MCSI and duration of illness ((r=-0.089, p=0.321).

Caregiving can be “direct” and includes helping patients with activities such as toileting, bathing, grooming, lifting, and giving home-based physical therapy. “Indirect” caregiving includes activities such as providing financial support and doing household chores [20]. In our study, the majority of patients were feeling overwhelmed due to caregiving. “Feeling overwhelmed is arduous burdensome discomfort with perplexing immobilization surfacing with fervently pursuing repose” [29]. Caregiving in stroke is full-time work [5]. Being overburdened is difficult and uncomfortable [29].

Caregivers of stroke survivors usually give up their leisure activities and have less social interaction or change or loss of employment due to caregiving roles [21,29,30]. In our study, the majority of caregivers felt confined, changed their personal plans, and made work adjustments. Loss of job and change in job were seen in 4.8% and 8% of caregivers, respectively. Many caregivers experience financial strain either sometimes or on a daily basis.

Limitations of the study

The limitation of our study is that patients were recruited from a single tertiary care institute. Mostly, patients were from a specific geographical area and culture. This limits the generalizability of results to populations of other geographical areas with different cultural practices. Therefore, multi-centric studies are needed on populations of different geographical areas with different cultural practices.

## Conclusions

Stroke is devastating for both stroke patients and their caregivers. Caregivers of stroke survivors experience high levels of caregiving burden. The findings in our study provide evidence that caregivers of stroke survivors experience significant levels of strain. The strain levels were significantly higher in the caregiving of male stroke survivors, fully dependent survivors, and survivors with hemorrhagic stroke. The caregiving strain was independent of the gender of caregivers. More robust and large-scale studies are needed to validate these aspects of caregiver strain. It is desirable to recognize them and manage them during the rehabilitation of stroke survivors.
